# Storage Life Prediction of Rubber Products Based on Step Stress Accelerated Aging and Intelligent Algorithm

**DOI:** 10.3390/polym15010157

**Published:** 2022-12-29

**Authors:** Xiaohui Guo, Xiaojing Yuan, Guangyong Liu, Genliang Hou, Ze Zhang

**Affiliations:** 1 Combat Support College, Rocket Force University of Engineering, Xi’an 710025, China; 2 Key Laboratory of Rubber and Plastic Materials and Engineering, Ministry of Education, Qingdao University of Science and Technology, Qingdao 266042, China

**Keywords:** step stress accelerated aging test, intelligent algorithm, rubber storage life, acceleration factor, improved Arrhenius equation

## Abstract

Compared with the constant stress accelerated aging test, the step stress accelerated aging test reduces the accelerated aging test time by increasing the aging temperature step by step to obtain the aging failure life of rubber in a shorter time, but its data processing method is not mature enough. In this paper, a simplified step is proposed to process the step stress accelerated aging data. The identification of the acceleration factor is transformed into an optimization problem to avoid the error accumulation problem caused by fitting the data at each temperature. Considering the non-Arrhenius phenomenon in the rubber aging process, a modified Arrhenius equation was used to extrapolate the acceleration factor at low temperatures to calculate the prediction curves for the degradation of polyurethane rubber properties at low temperatures. The life prediction results of the constant stress accelerated aging test and step stress accelerated aging test were compared, and the dispersion coefficient between the two results was between 0.9 and 1. The results obtained by the two methods were in good agreement, which proved the correctness and feasibility of the method used in this paper.

## 1. Introduction

Since Charles Goodyear’s unintentional discovery of vulcanized rubber in 1844, rubber products have been widely used in engineering, such as tires, air springs, conveyor belts, and various sealing elements. As polymer materials, rubber materials, and rubber products gradually undergo physical or chemical changes in their structure and composition during storage or use due to the combined effects of heat, oxygen, light, humidity, ozone, and other external factors, and their properties gradually deteriorate leading to eventual failure, a phenomenon known as rubber degradation or aging [1,2]. If rubber products are not replaced before they fail, it often leads to sudden equipment failure and even serious safety accidents. Therefore, how to accurately predict the life of rubber products is of high engineering value in terms of improving the overall safety and reliability of equipment.

In the natural environment, the service life of most rubber products is between 3–25 years, which makes it difficult to predict their service life at the design stage, and it will take a lot of time to observe their life by using natural aging, which is obviously unrealistic. The time-temperature equivalence principle means that the rate of rubber aging reaction is proportional to the aging temperature, and the time to reach failure will be shortened with the increase of temperature [3,4,5]. Based on this, an accelerated aging test is proposed to be widely used for the life prediction of rubber products.

The accelerated aging test is divided into constant stress accelerated aging test, step (decreasing) stress accelerated aging test, and sequential stress accelerated aging test. The most mature one is the constant stress accelerated aging test, but its disadvantage is that it takes longer time, and the step (decreasing) stress accelerated aging test takes less time, but its data processing method is not mature enough. The core of the accelerated aging test is the identification of the acceleration factor (also called translation factor). So far, most of the accelerated aging life predictions still use the traditional data processing method, i.e., fitting the aging data at different temperatures to obtain the aging life at each temperature and then extrapolating the aging life at the target temperature [6,7,8,9]. The disadvantage of the traditional method is that the data at each temperature will bring the problem of superposition of errors, which will get less accurate acceleration factors and lead to poor accuracy of the final life prediction results. Therefore, it is particularly important to identify the acceleration factors in the accelerated aging process accurately, because it directly determines the accuracy of the life prediction [10,11,12].

The emerging intelligent algorithm has better applicability in parameter identification, and its application to the life prediction of rubber products can avoid the multiple fitting of accelerated aging data at each temperature, which effectively avoids the resulting error superposition problem and improves the accuracy of the acceleration factor identification results.

In this paper, the accelerated aging test of polyurethane rubber, the main material of flexible oil tank, is carried out by step stress accelerated aging test; the accelerated factors at each aging temperature are coded as vectors; and the artificial bee colony algorithm is used to obtain the optimal solution. The regression model of acceleration factor versus temperature was established by the improved Arrhenius equation and extrapolated to the acceleration factor at low temperature and then the predicted aging performance decline curve was obtained. The predicted results were compared with those of the constant stress accelerated aging test, and the results obtained by the two test methods were in good agreement, which proved the accuracy and feasibility of the proposed method in the data processing of the step stress accelerated aging test.

## 2. Accelerated Aging Test Model and Principle

### 2.1. Step-Stress Accelerated Aging Test

**Figure 1:**
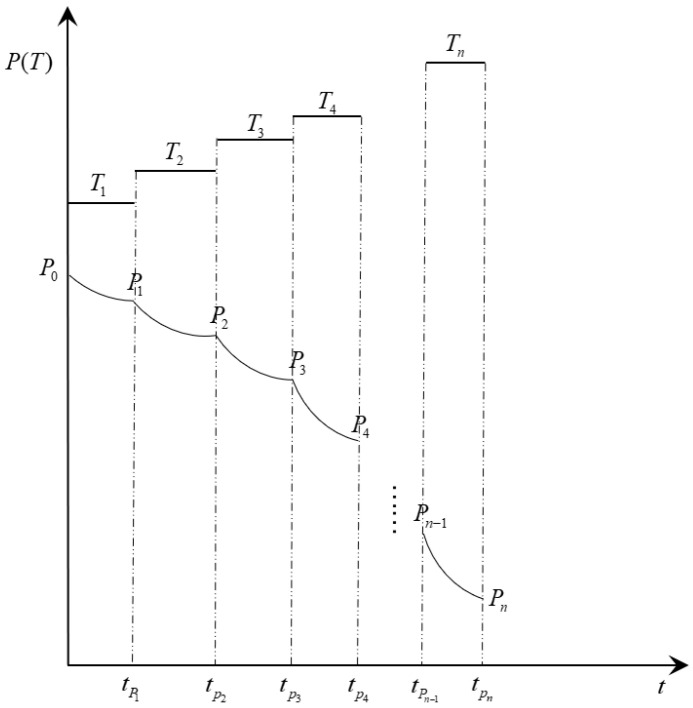
Schematic diagram of step stress loading and performance degradation.

Compared to the constant stress accelerated aging test, the step stress accelerated aging test only requires one set of tests to complete the life prediction of the specimen. Assume that there are n stress levels, $T_{1},T_{2},T_{3},$ …, $T_{n}$, and the critical value of performance index at the end of each stress is $P_{1},P_{2},P_{3},$ …, $P_{n}$, the initial performance index value is $P_{0}$, and the time to reach the critical value of performance index is $t_{P_{1}},t_{P_{2}},t_{P_{3}},$ …, $t_{P_{n}}$, respectively, as shown in Figure 1.

The step stress accelerated aging test requires the stress to be adjusted to the next set value when the aging performance index drops to a predetermined value. The test conducted in this paper specifies that the stress is adjusted to the next stress level when the aging performance index drops by 10% of the initial aging performance index at the stress level, and the temperature is used as the stress variable in the step stress accelerated aging test.

The rubber studied in this paper is polyurethane rubber, the main material of flexible oil tank. According to the working principle of the flexible oil tank, the tensile strength retention rate is a characteristic directly related to sealing performance and reflects the performance of the flexible oil tank, so the tensile strength retention rate is chosen as the performance decline index of the accelerated aging test. The data of step accelerated aging of polyurethane rubber in the literature are used as an example to verify the validity of the improved Arrhenius model proposed in this paper. Table 1 shows the performance degradation data of the step stress accelerated aging of polyurethane rubber, and the performance degradation index is the tensile strength retention rate. The test was carried out at four incremental step temperatures, with a step temperature change time of 10% of the performance degradation, and the temperature was increased from 70 °C to 110 °C in sequence, and finally the performance degradation was stopped at 60% of the initial value.

Use a dumbbell II test cutter and rubber punching machine to intercept dumbbell-shaped specimens on polyurethane rubber specimens, suspend the specimens in the high and low-temperature test chamber, set the aging temperature to 70 °C, start timing, take the test cycle for 1 d, at the test time node, take out part of the specimens (not less than 3), in accordance with GB/T 2941 “rubber test environment regulation and test standard temperature, humidity and time” for environmental conditioning, in accordance with HG/T2580-2008 (rubber or plastic coated fabric tensile strength and elongation at break determination) to complete the tensile test, and take the average of the tensile strength retention rate as the test data. When the decrease of tensile strength retention rate reaches 10%, set the temperature stress level to 82.4 °C, restart the timing, and continue the accelerated aging test on the polyurethane rubber, and so on, until the temperature stress level reaches 110 °C and the tensile strength retention rate decreases to 40%, then stop the test.

### 2.2. Principle of Time-Temperature Equivalent Superposition

Since the Arrhenius equation was proposed, it has been widely used in the field of life prediction of rubber products, and a large number of studies have shown that the variation law of the aging rate of most rubber materials with temperature can be described by the Arrhenius equation [3,4,5]. However, recent studies have shown that the activation energy of rubber products during aging changes when the aging temperature spans a large range, when the activation energy is assumed to be a constant that does not change with temperature, the extrapolation life obtained has a large error with the actual life [13], which is known as a non-Arrhenius phenomenon [14], and the traditional Arrhenius equation will no longer be applicable in some cases [13,15], so the introduction of power exponential factor has improved the traditional Arrhenius equation [16].

(1) $$k(T)=Ae^{-{\left(\frac{E_{b}}{RT}\right)}^{n}}$$

where k(T) represents the aging reaction rate at aging temperature, A is a temperature-independent constant, $E_{b}$ is the actual activation energy of the aging reaction, and R is the ideal gas constant. The logarithmic transformation of Equation (1) gives the following form:

(2) $$Ln(k)=a+b/T^{n}$$

The slope of the Ln(k)−1/T curve can be obtained from the derivative of Equation (5) with respect to 1/T. Multiplying this slope by −R, the equivalent linear activation energy is obtained as:

(3) $$E_{a}=-nbR/T^{n-1}$$

The activation energy in the aging process derived from the conventional Arrhenius equation is $E_{a}=-bR$. Comparing the activation energy in the aging process derived from the two equations, the activation energy obtained from the modified Arrhenius equation is a function of temperature, while the activation energy obtained from the conventional Arrhenius equation is a constant, and it is obvious that the modified Arrhenius equation is more consistent with the recent research results.

Define the acceleration factor as the ratio of the aging reaction rate at any aging temperature to the aging rate at the reference temperature.

(4) $$a\left(T\right)=k\left(T\right)/k\left(T_{0}\right)=e^{{\left(\frac{E_{b}}{RT_{0}}\right)}^{n}-{\left(\frac{E_{b}}{RT}\right)}^{n}}$$

where a(T) is the acceleration factor corresponding to temperature T, $k\left(T\right),k\left(T_{0}\right)$ are the aging reaction rates at T and reference temperature, respectively. After obtaining the acceleration factor at a high temperature, the acceleration factor at low temperature can be obtained by extrapolation using Equation (7).

The relationship between the aging performance decline index P and time t of rubber can be described by a P-t binary mathematical model, given an aging temperature T, the aging performance decline law of rubber is as in Equation (5) [17].

(5) $$P=Ae^{-k\left(T\right)t^{\alpha }}$$

where A,α is a constant independent of time and k is the aging reaction rate. Substituting Equation (4) into Equation (5):

(6) $$P=Ae^{-k\left(T_{0}\right){\left(\sqrt[{\alpha }]{a\left(T\right)}t\right)}^{\alpha }}$$

Therefore, when the aging time of rubber at temperature T is t, its equivalent aging time at the reference temperature is $\sqrt[{\alpha }]{a\left(T\right)}t$. The equivalent aging time of any aging temperature to the reference temperature can be obtained as:

(7) $$t=\sqrt[{\alpha }]{a\left(T\right)}t\left(T\right)$$

Equivalent aging time at any temperature relative to the reference temperature can be obtained by Equation (7).

**Table 1:** Tensile strength retention at different aging temperatures [18].

Performance (%)	Aging Time									
	1	2	3	4	5	6	7	8	9	10
70 °C	100.8	98.8	97.5	95.7	94.1	93.4	92.8	91.6	90.3	90.0
82.4 °C	88.8	87.0	85.4	84.6	84.0	82.6	81.2	80.8		
95.6 °C	78.6	77.3	76.3	74.5	72.8	72.1	71.4			
110 °C	68.7	66.2	64.8	62.7	61.6	60.6				

## 3. Acceleration Factor Identification Based on the ABC (Artificial Bee Colony) Algorithm

### 3.1. Acceleration Factor Identification Process

The proposed method considers the acceleration factor a(T) at different aging temperatures as a candidate solution for the artificial bee colony algorithm, and solves the optimal solution by the artificial bee colony algorithm for the aging life prediction at low temperatures.

For simplicity of description, the description of the data needs to be declared in advance. The aging performance index at each aging temperature is called $P_{ij}$, the aging moment at each aging temperature is called $t_{ij}$, i=0,1,2,…,n, where n is the number of aging temperatures, j=0,1,2,…,m, where m is the number of tests at each aging temperature, and the number of tests at different aging temperatures will vary.

In the step stress accelerated aging test, except for the initial aging temperature, the initial values of the aging performance indexes under the aging temperature are the values at the end of the loading of the previous aging temperature, so the test data under these temperatures need to be shifted. Assume that the initial performance index of aging at the reference temperature is $P_{0}$, the initial performance index of aging at temperature $T_{i}$ is $P_{i0}$, the aging performance index at reference temperature decreases from $P_{0}$ to $P_{i0}$ in time $t_{i}$, and the aging performance index at temperature $T_{i}$ decreases from $P_{i0}$ to $P_{ij}$ after time $t_{ij}$. At this point, the equivalent aging time of aging performance index from $P_{0}$ to $P_{ij}$ under temperature $T_{i}$ relative to the reference temperature is:

(8) $$t=t_{i}+\sqrt[{\alpha }]{a_{i}}t_{ij}$$

The test data at the reference temperature are fitted by Equation (5), which in turn calculates $t_{i}$. The data at each aging temperature are shifted to the reference temperature by Equation (8), and the regression analysis is performed on the shifted data using the idea of least squares, where the regression coefficient is estimated so that the total sum of squared residuals is minimized at which point f(x) is a function of a(Τ):

(9) $$f\left[a\left(T\right)\right]=\sum_{i=1}^{n}\sum_{j=1}^{m}{\left({\hat{P}}_{ij}-P_{ij}\right)}^{2}$$

**Figure 2:**
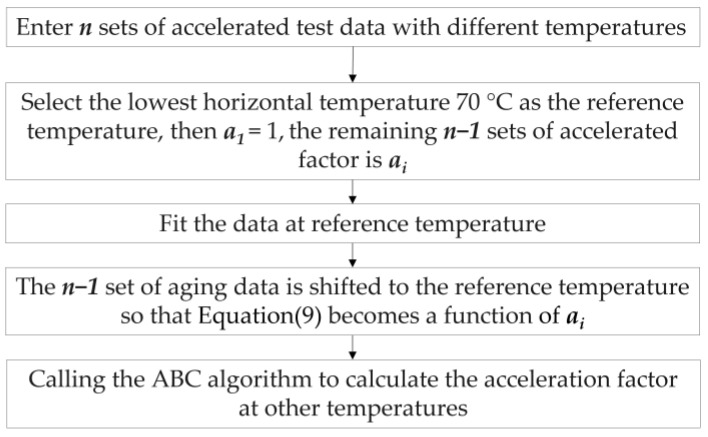
Steps to accelerate factor identification.

The problem is transformed into the optimal solution for the minimal value of Equation (9), and Equation (9) is used as the objective function of the artificial bee colony algorithm or other intelligent algorithms to be solved to obtain the optimal solution of the acceleration factor. The specific steps of the step stress accelerated aging test acceleration factor identification are shown in Figure 2.

### 3.2. Acceleration Factor Identification Results

**Figure 3:**
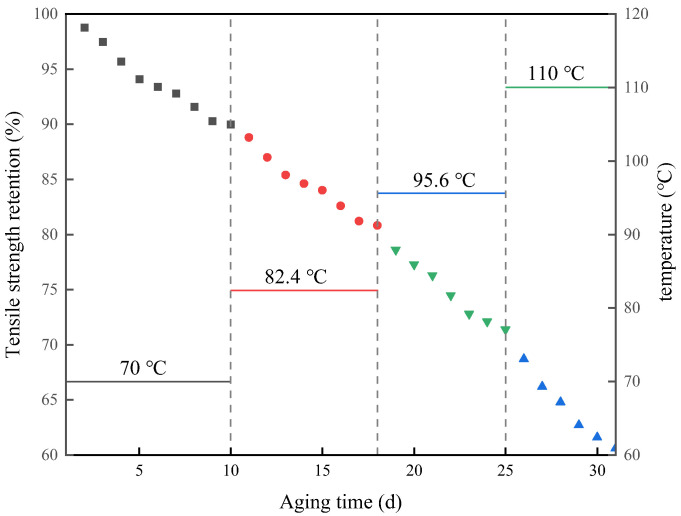
Changes in tensile strength retention with time.

Table 1 shows the tensile strength retention under the step accelerated aging test of polyurethane rubber, and the step stress accelerated aging temperatures were 70 °C, 82.4 °C, 95.6 °C, and 110 °C, respectively, with the loading method shown in Figure 3. Using 70 °C as the reference temperature for accelerated factor identification, the kinetic equation is fitted to the data at 70 °C by Equation (5), the genetic algorithm is used in this process to identify α, and the fitting equation is shown in Equation (10).

(10) $$y=1.056e^{-0.046t^{0.5506}}$$

Combining Equation (10) with Equation (8) we can get the performance degradation curve of polyurethane rubber at any temperature as:

(11) $$y=1.056e^{-0.046{\left(t_{i}+\sqrt[{\alpha }]{a_{i}}t_{ij}\right)}^{0.5506}}$$

**Figure 4:**
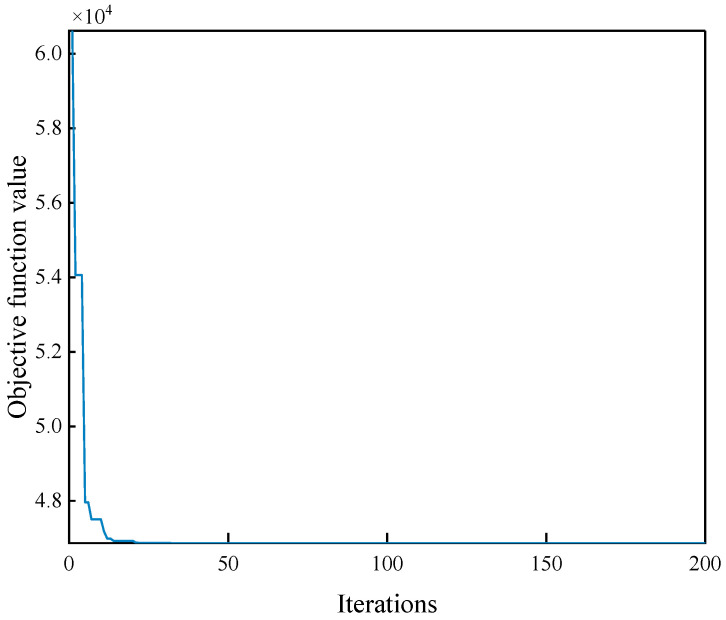
Convergence of the objective function with the number of iterations of the algorithm.

The aging data at the remaining temperatures are leveled to the reference temperature (70 °C) by Equation (8), and Equation (9) is used as the objective function of the artificial bee colony algorithm to solve for the acceleration factor at each temperature using the idea of least squares. Figure 4 shows the convergence of the objective function value during the iteration of the algorithm, and the final objective function value is 0.00047. 

**Figure 5:**
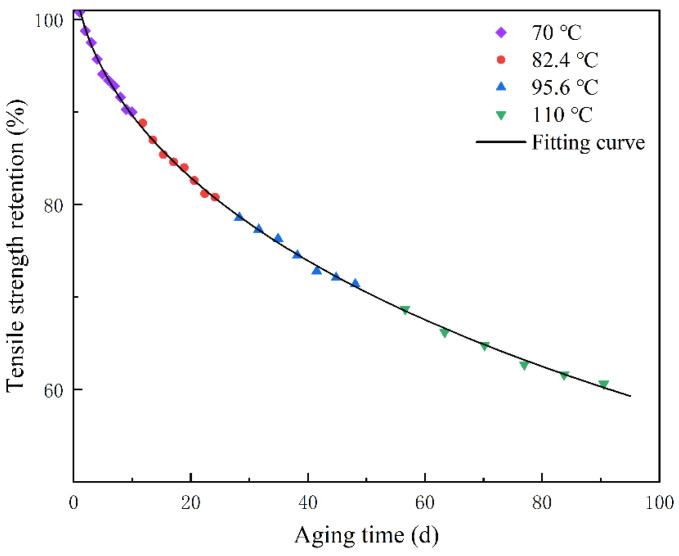
Aging data and nonlinear regression after time–temperature equivalent superposition.

**Figure 6:**
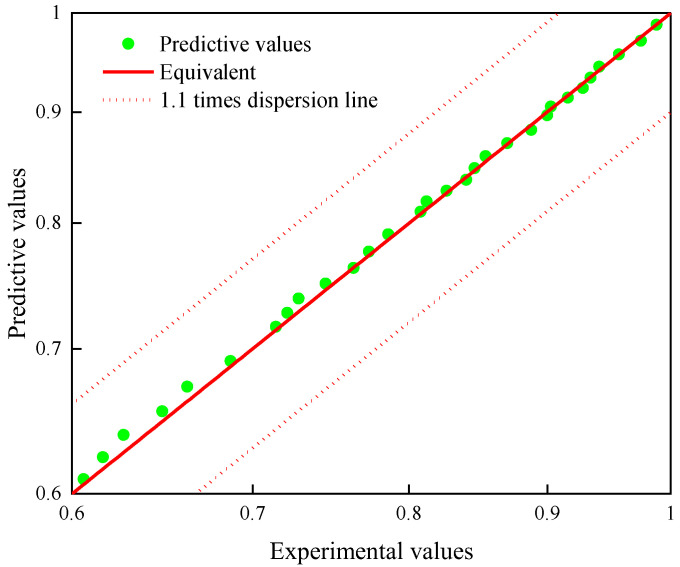
Dispersion map of regression predicted values and raw data.

Table 2 shows the acceleration factors at each aging temperature identified by the artificial bee colony algorithm, and the aging data at 82.4 °C, 95.6 °C, and 110 °C, three temperatures, were leveled to the reference temperature according to Equation (8), and Figure 5 shows the result of translating the aging data of other temperatures to the reference temperature. The data after translation are smooth and evenly distributed on both sides of the fitted curve. Figure 6 shows the dispersion between the predicted and measured values. The predicted values are evenly distributed on both sides of the measured value contour, and they are all within 1.1 times of the dispersion line, which indicates that the predicted results have good accuracy.

## 4. Prediction of Storage Life of Polyurethane Compounds

On the basis of accurate identification of the high temperature acceleration factor, a nonlinear regression analysis of the obtained acceleration factor is performed by using the idea of least squares method by Equation (4) in which a genetic algorithm is used to identify the parameter n and obtain n=2.0023, the regression equation is:

(12) $$a=e^{{\left(\frac{6172.4}{8.314\times 303.15}\right)}^{2.0023}-}{}^{{\left(\frac{6172.4}{8.314\times T}\right)}^{2.0023}}$$

**Figure 7:**
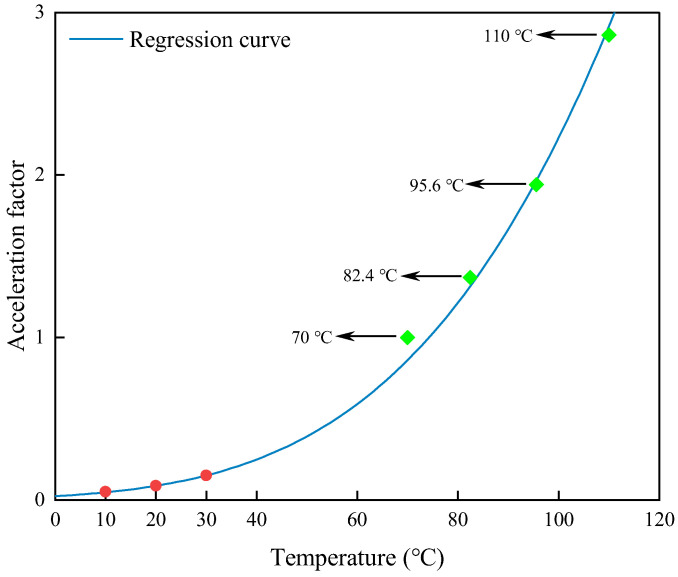
Regression and extrapolation results of the acceleration factors.

**Figure 8:**
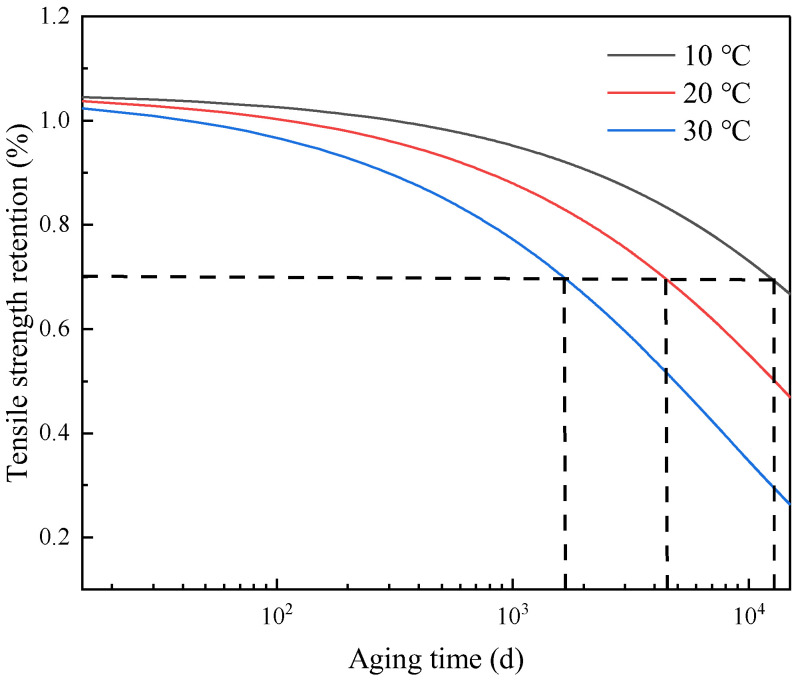
Performance degradation prediction curves at different temperatures.

The acceleration factor at low temperature was extrapolated by Equation (12), as shown in Figure 7, and the acceleration factor at low temperature was obtained by extrapolation as shown in Table 3. In order to verify the accuracy of the acceleration factor at low temperature, the acceleration factor at low temperature was substituted into Equation (12), and the performance degradation prediction curve of the polyurethane at low temperature was obtained, as shown in Figure 8.

It is assumed that the tensile strength retention rate of the polyurethane adhesive decreases to 70% and the failure life at 10 °C, 20 °C and 30 °C is calculated according to the prediction curve of the performance degradation of the polyurethane adhesive at low temperature and compared with the prediction result of the constant stress accelerated aging test, which is shown in Table 4. The dispersion coefficient is between 0.9 and 1, which verifies the accuracy of the improved Arrhenius model proposed in this paper.

## 5. Conclusions

This paper introduces the use of intelligent algorithms to accurately identify the acceleration factors for rubber step stress accelerated aging tests. The acceleration factors at different aging temperatures are used as candidates for the artificial bee colony algorithm, making the identification of acceleration factors an optimization problem. The sum of squared residuals is used as the objective function of the artificial bee colony algorithm, so that the matching quality between the identification results and the test results can be evaluated and the optimal acceleration factor value can be finally found. The intelligent algorithm for solving the acceleration factor avoids the error accumulation problem caused by fitting the aging data at each temperature, which can improve the accuracy of the final acceleration factor identification results and greatly simplify the process of solving the acceleration factor.

The acceleration factor obtained by the artificial bee colony algorithm is used to shift the test data at each aging temperature to the reference temperature, and the residual sum of squares of the aging performance degradation curve between the shifted data and the reference temperature is 0.00047, which proves that the intelligent algorithm identifies the acceleration factor with high accuracy.

The improved Arrhenius equation was introduced to extrapolate the acceleration factor at the used temperature, and the prediction curve of rubber aging at low temperature was obtained. The prediction curve can well predict the aging of rubber at room temperature, which proves the feasibility of the artificial bee colony algorithm in the process of acceleration factor identification.

The predicted results of the step stress accelerated aging test were compared with the predicted results of the constant stress accelerated aging test, and it was found that the dispersion coefficient between the predicted life obtained by the method of this paper and the predicted results of the constant stress accelerated aging test was in the range of 0.9-1 with good agreement. This proves the correctness and feasibility of the proposed method in step stress accelerated aging experimental data processing because it improves the efficiency of data processing and the accuracy of prediction results on the one hand and reduces the accelerated aging test time on the other hand.

## Figures and Tables

**Table 2:** Identification results of rubber aging acceleration factors at different temperatures.

Temperature	70 °C	82.4 °C	95.6 °C	110 °C
Acceleration factor	1.00	1.37	1.94	2.86

**Table 3:** Acceleration factor extrapolation results at low temperature.

Temperature	10 °C	20 °C	30 °C
Acceleration factor	0.0502	0.0886	0.1517

**Table 4:** Comparison of life prediction results between traditional and improved Arrhenius equation.

Temperature	Aging Life (Year)		Dispersion Coefficient
	Constant Stress Prediction Results [18]	Step Stress Prediction Results	
10 °C	30.6	33.1	0.92
20 °C	11.4	11.7	0.97
30 °C	4.5	4.4	0.98

## Data Availability

The data presented in this study are available on request from the corresponding author.
